# A decade of norovirus disease risk among older adults in upper-middle and high income countries: a systematic review

**DOI:** 10.1186/s12879-015-1168-5

**Published:** 2015-10-14

**Authors:** Lisa Lindsay, Joanne Wolter, Ilse De Coster, Pierre Van Damme, Thomas Verstraeten

**Affiliations:** P95 Pharmacovigilance and Epidemiology Services, Leuven, Belgium; Contractor to P95 Pharmacovigilance and Epidemiology Services, Brisbane, Australia; University of Antwerp, Antwerp, Belgium

**Keywords:** Norovirus, Older adults, Gastroenteritis, Epidemiology, Risk, Disease burden

## Abstract

**Background:**

Noroviruses (NoVs) are the most common cause of acute gastroenteritis (AGE) causing both sporadic and outbreak-associated illness. Norovirus (NoV) infections occur across all ages but certain sub-groups are considered at increased risk due to heightened transmission and/or symptom severity. Older adults are potentially at high risk of NoV-associated illness due to frequent outbreaks in long-term care facilities (LTCFs) and severe health outcomes following infection. Elucidation of NoV risk among older adults will support prevention, treatment and control efforts.

**Methods:**

We conducted a systematic literature review to summarize the published risk estimates of NoV-associated illness, hospitalization and death among individuals aged 65 years and older. A structured search using defined NoV and gastroenteritis (GE) terms was performed in the PubMed and EMBASE databases of human studies published between January 1, 2003 and May 16, 2013.

**Results:**

We identified 39 studies from high income (HI) and upper-middle income (UMI) countries. Thirty-six percent of publications provided risk estimates based on laboratory-confirmed or epidemiologically-linked population-based surveillance data using molecular diagnostic methods. Over the study period, estimated annual NoV rates and extrapolated number of cases among older adults in HI and UMI countries were: 29-120/10,000 or 1.2–4.8 million NoV-associated illnesses; 18–54/10,000 or 723,000–2.2 million NoV-associated outpatient visits; 1–19/10,000 or 40,00–763,000 NoV-associated inpatient visits; 0.04–0.32/10,000 or 2000–13,000 NoV-associated deaths. NoV was responsible for approximately 10–20 % of GE hospitalizations and 10–15 % of all-cause GE deaths among older adults. Older adults experienced a heightened risk of nosocomial infections. Those in LTCFs experience frequent NoV outbreaks and the range in attack rates was 3–45 %, case hospitalization rates 0.5–6 % and case fatality rates 0.3–1.6 %.

**Conclusions:**

Older adults are at increased risk of severe NoV-associated health outcomes. NoV-associated hospitalization rates were higher, more severe, resulted in longer stays and incurred greater costs than for younger patients. NoV-associated mortality rates were approximately 200 % higher among individuals 65 years and older compared to <5 years. The burden of NoV among older adults is expected to rise along with societal aging and increased need for institutionalized care. NoV prevention in older adults, including potential vaccination, may significantly impact risk of severe illness.

## Background

Noroviruses (NoVs) are non-enveloped, single-stranded RNA viruses that cause both sporadic and epidemic illness. NoVs are the most common cause of acute gastroenteritis (AGE) in both adults and children and are characterized by an incubation period of 1–2 days and acute onset of non-bloody diarrhea, nausea, vomiting and abdominal cramps with possible fever, malaise and anorexia [1–5]. NoVs are grouped into at least five genogroups based on the viral capsid gene and within each genogroup up to 20 genotypes have been identified [6]. Genogroups I and II are the most common in humans with genotype GII.4 accounting for the majority of norovirus (NoV) cases globally, due primarily to GII.4 2012 Sydney and 2009 New Orleans strains in recent years [2, 7–15].

NoV infections are extremely common and start to occur early in life. In a Peruvian birth cohort approximately 80 % of NoV infections occurred within the first year of life [16]. By adulthood, 90 % of individuals in both England and Chile have detectable antibodies against NoV [1]. While there is evidence of both innate and acquired genotype-specific (but not genogroup) immunity, there is some controversy over the specificity and duration of NoV immunity as well as the role of asymptomatic infections [1, 16–20]. NoV evolution occurs through adaptive changes in the capsid P2 domain, particularly for genotype GII.4, which allows the virus to escape herd immunity [12, 17].

NoV AGE outbreaks are extremely common due to viral evolution, low infectious dose, extended viral shedding, sustained environmental persistence, and low background immunity [6, 21–25]. NoV has distinct seasonal activity with 75 % of all cases occurring during cooler months and annual fluctuations in the number and severity of cases largely due to the emergence of novel genotypes or variants [26]. NoV is the leading cause of foodborne illness responsible for approximately 60–65 % of such illness in both the United States (US) and Canada as well as being the second leading cause of foodborne hospitalizations (26 %) following non-typhoidal *Salmonella spp* (35 %) [4, 27]. While foodborne NoV outbreaks are well documented, the most frequent setting for outbreaks is healthcare facilities (including both hospitals and long-term care facilities (LTCFs)) in which person-to-person transmission of genotype GII.4 is most common [2, 6, 28–38]. NoV GII.4 is responsible for the majority of NoV cases in both adults and children, however the relative importance of specific GII.4 variants and non-GII.4 genotypes may vary according to outbreak setting (GII.4 New Orleans and Sydney predominate in healthcare settings) and transmission route (GI.3, 6, 7 and GII.3, 6, 12 predominate in foodborne outbreaks) [7, 8, 11]. Furthermore, in a systematic review NoV GII.4 was reported to be associated with more severe outcomes than other genotypes in all ages [33].

While NoV causes sporadic and outbreak AGE in all age groups, specific sub-groups may be at heightened risk due to increased risk of exposure and subsequent illness and/or heightened risk of severe outcome once infected. Such populations include the very young and old, individuals who are immunocompromised, and those living in closed/semi-closed communities [1, 5, 39, 40]. The pediatric population experiences an extremely high incidence of both non-medically attended and medically-attended NoV illness. Recent reports indicate that, since the introduction of vaccination against rotavirus disease, which has decreased the burden of this childhood illness, NoV has become the leading cause of pediatric AGE and hospitalizations in diverse settings such as the US, Finland and Nicaragua [41–48]. There is also evidence to suggest that older adults may be at increased risk of severe NoV illness [49–51]. This may be due to intrinsic factors such as age-related alterations in B and T cell function as a result of immunosenescence or the presence of comorbidities (such as immunosuppression, renal disease, cardiovascular disease and/or functional disability) that can reduce an individual’s ability to mount a successful immune response to infection and result in more severe/extended symptoms or exacerbation of underlying conditions [49, 52, 53]. Extrinsic factors such as institutionalized housing may further influence exposure potential or be a proxy for poor underlying health status [33, 54–57]. Determining the importance of NoV as a causal pathogen and estimating the NoV-associated risk among older adults is important for prevention efforts.

Development of vaccines against NoV is currently underway, but there are no approved NoV vaccines to date [18, 58–62]. As a frequent cause of AGE in all age groups, prevention of NoV illness through vaccination could have a substantial impact on the worldwide burden of sporadic and outbreak AGE. Estimates of the disease burden among at-risk populations, such as older adults, will help target clinical development of novel vaccines and inform future vaccination policy decisions. We conducted a systematic literature review to determine the risk of NoV-associated gastroenteritis (GE), hospitalization and death in older adults.

## Methods

### Search strategy and selection criteria

We conducted a literature search within PubMed and Embase using the following MESH (Medical Subject Headings) terms and the free text term ‘Norwalk-like disease’: ((norovirus OR norwalk-like virus OR norwalk-like disease) AND epidemiology) OR (gastroenteritis AND (incidence OR prevalence OR surveillance OR epidemiology) NOT (Appendicitis OR Cholera Morbus OR Diverticulitis OR Esophagitis OR Inflammatory Bowel Diseases OR Mucositis OR Proctitis OR cancer OR children OR infant)). We did not limit our search to studies among older adults but reviewed all possible publications given the scarcity of data specific to this population and the inclusion of age-stratified results in community-based research. We limited articles to human studies published in English between January 1, 2003 and May 16, 2013, which had an abstract available. Publications were excluded if they described a single outbreak given that such publications, although informative, may produce less generalizable results due to outbreak specific conditions with respect to genotype circulation, population characteristics, outbreak reporting and control activities. Articles were reviewed for any information concerning NoV incidence, prevalence, attributable proportion and attack rates among older adults (individuals aged ≥65 years). Studies conducted exclusively in LTCFs were considered to include predominantly older adults. Other extracted information included study setting, NoV diagnostic method, and outbreak status (e.g., restriction of data to periods of heightened norovirus activity or inclusion of both outbreak and non-outbreak periods) at the time of the study. We considered studies by duration (1 year or >1 years duration) and availability of numerator and denominator data. We present data according to health outcome and type of risk estimate (incidence per 10,000 followed by attributable proportion for less specific GE) for studies that clearly identify NoV-specific estimates. Furthermore, we present data separately for studies that exclusively reported nosocomial cases and long-term care facility (LTCF) outbreaks.

We calculated the number of NoV GE cases in high-income (HI) and upper-middle income (UMI) countries for NoV outpatient visits, hospitalizations and deaths among older adults by extrapolating estimated incidence rates to cumulative HI and UMI country population figures for those ≥65 years based on 2013 World Bank population estimates (http://data.worldbank.org). HI countries were considered to be those countries with an annual gross national income per capita of ≥ $12,616 and UMI countries were considered to be countries with an annual gross national income per capita between $4086 and $12,615 per capita representing approximately 402 million individuals aged ≥65 years (209 million and 193 million in HI and UMI countries, respectively).

## Results

### Search results

**Fig. 1:**
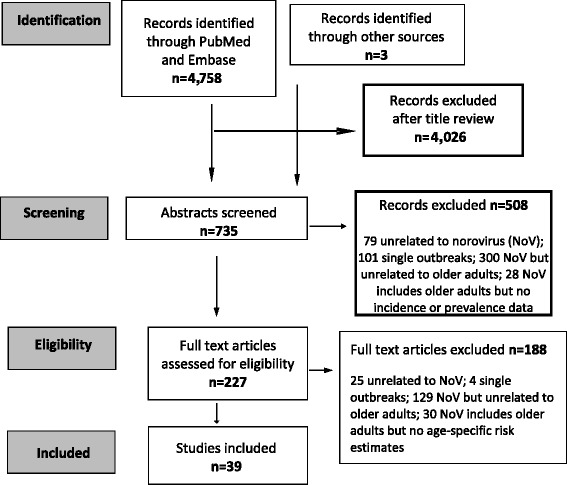
Literature search summary

A summary of the literature review process is given in Fig. 1. Publications that provided information on NoV risk by age among older adults are summarized in Table 1. We identified 39 studies conducted in 15 HI and UMI countries (44 % Europe, 28 % in the US/Canada, 23 % in Australia, Asia and the Middle East, and 5 % global reviews which were based predominantly in HI and UMI countries). Data were included in the identified studies from 1966–2013.

**Table 1:** Summary of 39 studies with norovirus (NoV) incidence or prevalence data among older adults

Author (Ref)	Country	Study years	Study design	Lab Method	Risk estimation method^a^	Age	Data source/population	Outcome of interest
Trivedi [89]	US	2009–2010	Retrospective cohort	NS^b^	C	NS	NORS and Medicare/LTCFs^c^ in 3 states	All cause hospitalizations and deaths during outbreaks
Hall [51]	US	1999–2007	Retrospective regression analysis	NS	C	All	National Center for Health Statistics/nationwide GE^d^ deaths	Incidence and attributable proportion of unspecified GE deaths
Shahid [109]	US	2007	Retrospective laboratory review	Elisa	B	All	In- and outpatient laboratory samples/city-wide patients tested for NoV	Characteristics of NoV positive specimens
Lopman [70]	US	1996–2007	Retrospective regression analysis	NS	C	All	National Inpatient Sample/nationwide GE hospital discharges	Incidence and attributable proportion of unspecified GE discharges
Rosenthal [38]	US	2003–2006	Prospective surveillance	PCR	A	18–106 y	AGE^e^outbreaks/LTCFs in 1 state	Attack rates, case-hospitalization and fatality rates during NoV outbreaks
Chui [71]	US	1991–2004	Retrospective database review	NS	D	65– <85 y	Medicare and Medicaid and US Census/nationwide hospitalizations	Incidence of specific gastrointestinal disease hospitalizations
Gastañaduy [45]	US	2001–2009	Retrospective regression analysis	NS	C	All	MarketScan Insurance Claims/US emergency and outpatient visits among insured	Incidence of emergency and outpatient visits for cause-specified and unspecified GE attributed to NoV
Leshem [35]	US	2010–2013	Retrospective analysis surveillance	PCR	A	All	Sentinel community NOV surveillance (NORS and CaliciNet)/community outbreaks in 5 states	Age-specific NoV distribution during GII.4 outbreaks
Leshem [110]	US	2010–2012	Retrospective analysis surveillance	PCR	A	All	Sentinel community NoV surveillance (NORS and CaliciNet)/community outbreaks in 27 states	Age-specific NoV distribution during GI.6 outbreaks
Hall [111]	US	1979–2010	Literature review	NS	E	All	Published studies with population-based incidence rates of NoV/US studies	Summary NoV incidence estimates
Ruzante [72]	Canada	2001–2004	Retrospective database review	NS	D	All	Canadian Institute for Health Information, Vital Statistics Registry, National Notifiable Diseases database/nationwide hospitalizations and deaths	Age-specific hospitalization and mortality incidence rates based on NoV-specific diagnostic coding
Friesema [87]	NL	2005–2007	Prospective surveillance	PCR	A	42–100 y	NoV outbreaks/LTCFs with nursing wards in 6 local health services	Characteristics of NoV outbreaks by genotype and symptoms
Van Asten [77]	NL	1999–2007	Retrospective analysis surveillance	NS	C	≥65 y	Statistics Netherlands (mortality), weekly laboratory surveillance/nationwide unspecified GE	Attributable proportion of all-cause deaths
Van Asten [68]	NL	1994–2006	Retrospective analysis surveillance	NS	C	≥65 y	Netherlands Information Network of GPs, NoV surveillance, National Medical Register, Statistics Netherland/nationwide unspecified GE	Incidence and attributable proportion of unspecified GE deaths
Verhoef [80]	NL	2009	Retrospective analysis surveillance analysis	NS	C	All	SENSOR study, German notifiable disease data, other literature/nationwide	Age-specific mortality, disease burden and burden due to foodborne NoV GE
Spackova [82]	Germany	2002–2008	Retrospective analysis surveillance	NS	A	All	Hospitalization reports: German notifiable disease data/nationwide	Nosocomial GE due to enteric pathogens including NoV
Werber [63]	Germany	2004–2008	Retrospective analysis surveillance	PCR	A	All	German notifiable disease data, Federal Statistical Office/nationwide	Incidence, deaths and potential life lost due to pathogen-specific GE
Bernard [32]	Germany	2001–2009	Retrospective analysis surveillance	PCR	A	All	German notifiable disease data, Federal Statistical Office/nationwide	Age-specific incidence, hospitalization and mortality due to NoV
Gustavsson [55]	Sweden	2008–2009	Retrospective case–control	PCR	B	≥60 y	Hospital database/hospitalized adults at one hospital	30 and 90 day survival rates
Fernandez [65]	Spain	2000–2007	Retrospective laboratory-based	PCR	B	All	Patient laboratory stool samples	Age-specific prevalence of enteric pathogens in GE cases
Arias [112]	Spain	2004–2005	Prospective surveillance	PCR	A	All	Mandatory physician reported outbreaks/Catalonia region	Age-specific incidence of GE
Manso [67]	Spain	2010–2011	Prospective laboratory-based	PCR	B	All	In- and outpatient laboratory stool samples/hospital complex	Age-specific prevalence of enteric pathogens in GE cases
Huhulescu [64]	Austria	2007	Prospective cohort	PCR	A	All	Outpatients/3 physician clinics	Pathogen-specific prevalence in GE
Harris [78]	England/Wales	2001–2006	Retrospective regression analysis	NS	C	≥65 y	Community pathogen surveillance; mortality statistics: Health Protection Agency, Office of National Statistics/England and Wales	Attributable proportion of GE deaths
Haustein [69]	England	2000–2006	Retrospective regression analysis	NS	C	≥18 y	Hospital Episode Statistics and LabBase Health Protection Agency/nationwide	Incidence and attributable proportion of hospital admissions
Philips [42]	England	1993–1996	Prospective cohort	PCR	A	All	Outpatients/70 GP clinics nationwide	Age-specific NoV incidence
Philips [113]	England	1993–1996	Prospective cohort	PCR	A	All	Community and outpatient non-GE patients/cohort and 70 GP clinics nationwide	Prevalence of asymptomatic NoV
Rovida [76]	Italy	2011–2012	Retrospective laboratory-based	PCR	B	All	Inpatient laboratory samples (stool)/one hospital	Pathogen-specific prevalence of GE
Kirk [83]	Australia	2002–2008	Retrospective surveillance analysis	NS	A	Adults	GE outbreaks OzFoodNet/LTCFs nationwide	Pathogen-specific GE outbreak incidence and characteristics of GE outbreaks
Davis [88]	Australia	2004–2007	Retrospective surveillance analysis	PCR	A	NS	GE outbreaks OzFoodNet/LTCFs in Queensland	Characteristics of NoV outbreaks in LTCFs
Tian [66]	China	2008–2009	Prospective cohort	PCR	B	≥14y	Outpatients with AGE/GE Department one hospital	NoV prevalence by age
Chan [74]	Hong Kong	2012–2013	Prospective cohort	PCR	B	All	Inpatients with NoV+ AGE/one hospital	NoV GII.4 distribution by age
Ho [37]	Hong Kong	2001–2006	Prospective laboratory-based analysis	PCR	A	All	Laboratory stool samples from inpatient, outpatients and outbreak cases with AGE/nationwide	Characteristics of NoV outbreaks by age, genotype and season
Tseng [114]	Taiwan	2005–2007	Prospective surveillance	PCR	B	>15 y	AGE cases at residential psychiatric care institution/one care center	Pathogen-specific GE hospitalization rate
Tang [75]	Taiwan	2011–2012	Prospective cohort	PCR	B	All	AGE symptomatic and asymptomatic in-, out- and emergency patients/one hospital	NoV prevalence by AGE symptom status
Lim [15]	Singapore	2004–2011	Retrospective laboratory	PCR	B	All	AGE stool samples/one hospital laboratory	Age-specific distribution of NoV positive samples
Al-Thani [73]	Qatar	2009	Prospective cohort	PCR	B	All	AGE emergency patients/one hospital	Enteric pathogen distribution by age
Greig [85]	Global	1997–2007	Literature review	NS	E	NS	GE outbreaks in LTCFs/global	Characteristics of enteric outbreaks by pathogen and transmission
Utsumi [86]	Global	1966–2008	Literature review	NS	E	NS	Infectious disease outbreaks in LTCFs/global	Characteristics of infectious disease outbreaks by pathogen

^a^Method: A = laboratory confirmed or epidemiologically-linked population-based surveillance; B = laboratory-confirmed clinic/hospital setting; C = indirect attribution from regression modeling; D = hospital database study select enteric illness codes without extrapolation E = literature review

^b^ *NS* not specified; NS diagnostic methods may have included molecular methods; NS age often reported for LTCFs generally serving older adults

^c^ *LTCF* long term care facility

^d^ *GE* gastroenteritis

^e^ *AGE* acute gastroenteritis

Risk estimates based on laboratory-confirmed or epidemiologically-linked population-based surveillance data were provided by 36 % (14/39) of studies, 28 % (11/39) were hospital or clinic-based laboratory-confirmed studies and 23 % (9/39) of the studies indirectly attributed NoV risk based on regression modeling. The remaining studies provided risk estimates based on secondary database review of NoV-specific hospitalization codes (5.1 %, 2/39) and literature reviews (7.7 %, 3/39). The included literature reviews diverged from the current study in scope of research interests and methods. NoV-associated outcomes reported in these studies included infections, illness, outpatient and emergency visits, hospitalizations and deaths. Ten primary studies reported NoV incidence data and 29 studies reported other measures of risk such as proportion of AGE cases positive for NoV or attack rates. Eleven publications focused predominantly on older adults. Seven publications (5 studies and 2 reviews) pertained exclusively to LTCFs (which did not always provide study-specific age distribution) and 1 publication pertained exclusively to nosocomial GE cases. NoV was detected using molecular methods in 22 studies, 1 study used immunoassay, and 16 studies used a variety of methods (which could have included molecular methods) or the method was not specified. Thirty studies considered study periods of at least 1 year.

### NoV-associated illness

No studies were identified that reported the community age-specific incidence of non-medically-attended NoV illness among older adults. German notifiable disease surveillance data reported community-level age-specific incidence of NoV-associated cases among individuals ≥65 years, which is presumed to include symptomatic individuals of varying severity [32, 63]. This data provided information on hundreds of thousands of individuals representing both sporadic and outbreak-associated community and institutional NoV cases that were laboratory or epidemiologically-confirmed [32, 63]. Age-specific mean incidence of NoV cases (2001–2009) demonstrated a U-shaped age distribution with the highest rates in the very young and older adults. NoV incidence (per 10,000) estimates from 2004–8 were approximately 54 in those <5 years, 5–15 in those 5–69 years, and 48 in those ≥70 years [63]. A second publication of the German surveillance data (2001–2008) provided a similar age-specific pattern with the highest incidence estimates per 10,000 in those <5 years (43) and individuals ≥75 years (28.9-119.8 depending on the 5-year age strata with highest estimates in those ≥85 years) [32].

### NoV-associated outpatient visits

Two studies report age-specific NoV-associated outpatient incidence estimates that include older adults [42, 45]. Both of these studies reported the highest rates in the very young. Model-based rates (per 10,000) from the US were 233 among those 0–4 years, 85 among those 5–17 years, 35 among those 18–64 years, and 54 among those ≥65 years [45]. Community-based age-specific incidence rates per 10,000 from England were 320 in those 0–4 years, 44 among 5–14 year olds, 38 among 15–44 year olds, 26 among 45–64 year olds and 37 among those ≥65 years [42].

Several publications report the mean proportion of outpatient GE visits due to NoV among middle-older aged adults (the lower age for many of these studies was 60 years) which ranged from 4–37 % [45, 64–67]. While most studies did not provide confidence intervals around these estimates, the proportion of GE outpatient visits due to NoV among older adults was not noticeably different from the estimated 6–26 % reported among other age strata [45, 64–67].

While the incidence of NoV-associated outpatient visits in older adults may not be as high as in the very young NoV outbreaks nevertheless result in large increases of unspecified GE outpatient visits in older adults [68]. In the Netherlands, the mean *monthly* incidence, per 10,000, of unspecified GE outpatient visits increased 70–240 % above average (15.0, 17.1 and 21.7 depending on the season) during NoV outbreaks with an overall mean of 9.1 among those ≥65 years. Taking the mid-point in the estimated proportion of outpatient GE visits due to NoV among older adults of 16.5 % (see above range estimates of 4–37 %), the Dutch monthly incidence data for unspecified GE outpatient visits can be extrapolated ((9.1*0.165)*12)) to an overall mean *annual* incidence per 10,000, of 18 with increased rates during NoV outbreaks up to 43.0 among those ≥65 years. These estimates depend substantially on the actual proportion of AGE due to NoV and the level/severity of NoV circulation. While it is expected that increased rates would occur across all age groups, a direct comparison by age was not possible due to the study’s focus on older adults.

### NoV-associated emergency department (ED) visits and hospitalizations

A single, US model-based study provided mean age-specific incidence estimates per 10,000 for NoV-associated ED visits which were: 38 among those <5 years, 10 among those 5–17 years, 12 among those 18–64 years, and 15 among those ≥65 years [45].

Four studies reported age-specific incidence rate estimates for NoV-associated hospitalizations among older adults [69–72]. Two of these studies were based on indirect model-derived estimates that accounted for under-diagnosis of NoV among hospitalized patient [69, 70] while the other two studies provided estimated NoV hospitalization rates based on NoV-specific diagnostic code(s) that did not include the estimated influence of NoV on non/unspecific GE diagnoses [71, 72].

English model-derived estimates of NoV-associated hospitalization records among adults estimated a rate range, per 10,000, of 0.23–0.48 inpatient admissions among 18–64 year olds and 1.0–4.3 among those ≥65 years [69] depending on the season. These estimates were based on national GE diagnostic codes and laboratory-based pathogen data. Higher estimates were obtained from a US model-based study that estimated the seasonal mean age-specific rate per 10,000 (and range) of NoV-associated hospitalization discharges as follows: 9.4 (5.3–18.5) among those <5 years, 1.1 (0.6–1.9) among 5–17 year olds, 1.0 (0.5–1.4) among 18–64 year olds, 4.7 (2.9–7.7) among 65–74 year olds, 9.2 (5.0–21.5) among 75–84 year olds, and 18.5 (10.6–37.9) among those ≥85 years [70].

Two studies reported estimated incidence rates for NoV-specific hospitalizations based on International Classification of Disease (ICD)-9 and 10 codes [71, 72]. While such specific estimates are considered underestimates of true NoV-associated hospitalization rates, they are useful for further burden of disease extrapolations and provide insight into variable diagnostic practices. One such study, a US-based Medicare and Medicaid Services study of individuals aged 65 to 85 years (representing >90 % of US elderly) reported an annual rate, per 10,000, of NoV-specific hospitalizations (based on ICD-9 code 008.63) of 0.004 among 65–85 year olds [71]. These authors highlighted that a substantial proportion of non-specific GE hospitalizations are likely to be undiagnosed viral GE in this population. Canadian-based national hospitalization data provided higher annual rates, per 10,000, of NoV-specific diagnosis (ICD-9 008.63 or ICD-10 A08.1) among middle aged and older adults (>59 years) of 0.61 [72]. These authors further noted that 63 % of NoV-specific hospitalizations were in this oldest age group.

Differences in the NoV hospitalization rate estimates between model-based indirect estimates [71, 72] and rate estimates based on NoV-specific diagnostic codes [69, 70], exceed 1000 fold when comparing the estimates of hospitalizations above 65 years by Chui et al. [71] to those for individuals 65–74 years reported by Lopman et al. [70]. These large differences highlight the extreme underestimation of risk based on pathogen-specific diagnostic codes. The validity of the model-based estimates is supported by seasonal peaks in non-specific GE hospitalization diagnoses that coincide with specific viral GE such as NoV.

The proportion of non-specific AGE ED/or hospitalized patients due to NoV among older adults has been reported in several model and laboratory-based studies [45, 69, 70, 73–76]. Qatar (laboratory-confirmed) and US-based (model derived) studies estimated that 12 and 17 % (respectively) of GE ED visits were due to NoV among adults aged >60 years [45, 73]. In England, >20 % of GE emergency hospital admissions were due to NoV among those ≥65 years during peak times of NoV activity [69].

The estimated proportion of hospitalized GE cases due to NoV among those ≥65 years, in model-derived US and English-based studies, was 8–13 % depending upon whether all-cause or unspecified caused GE hospitalization codes were included in the analyses [69, 70]. Approximately 50 % of hospitalized GE patients >65 years with stored stool samples were NoV-positive compared to approximately <20 % among younger age groups [76]. This higher estimate may reflect differences in the study population due to more select sampling of sick individuals with NoV-like symptoms, and may be less representative of a broad and diverse GE population.

NoV outbreaks are associated with large increases in unspecified GE hospitalizations in older adults. One study in the Netherlands demonstrated an increase in the mean monthly incidence of unspecified GE inpatient visits >30 % above average during NoV outbreaks (2.4, 2.7 and 2.6 per 10,000 depending on the season) with an overall mean of 1.9 among those ≥65 years [68]. Taking the mid-point in the estimated proportion of hospitalization GE visits due to NoV among older adults of 21.5 %, the Dutch *monthly* incidence data for unspecified GE inpatient visits can be extrapolated ((1.9 *0.215)*12)) to an overall mean *annual i*ncidence, per 10,000, of 4.9 with increased rates during NoV outbreaks up to 7.0 [68]. These estimates are comparable to those estimated in a US model-based study of approximately 5–19 per 10,000 [70].

NoV-associated hospitalizations in those ≥65 years have been reported to be more frequent, more severe, result in longer stays and require transfer to other care facilities following discharge compared to other age groups [32, 70, 72]. In the US, NoV-associated hospitalization rates and associated costs were highest among older adults, with half of the estimated $500 million per year spent on NoV-hospitalizations among those ≥65 years [70].

### NoV-associated mortality

Older adults contribute the vast majority of NoV-associated deaths [51, 55, 63]. US and German-based NoV-associated mortality rate estimates demonstrate a sharp increase with age [51, 63]. Specifically, US-based average annual mortality rate estimates, per 10,000, were 0.013 in those <5 years, 0.002 among those 5–64 years, and 0.20 among those ≥65 years (with fluctuations up to 55 % in this age group during non-epidemic and epidemic years) [51]. German NoV surveillance data reported similar NoV-associated mortality rate estimates per 10,000 namely, <0.01 in those <70 years to approximately 0.32 among those ≥70 years [63].

Dutch model-based estimates suggest that seasonal viruses account for nearly 5 % *of all-cause* mortality in the elderly with influenza accounting for the most deaths (approximately 2 %), followed by respiratory syncytial virus (1.5 %), parainfluenza (1 %) and NoV (0.2 %). The percentage of all deaths due to NoV increased with age to 0.5 % among those ≥85 years [77].

In the US, approximately half of all GE deaths (regardless of age) are coded as ‘cause unspecified’ of which older adults account for an estimated 83 % [51]. Among this population, *Clostridium difficile* is considered the leading cause of all-cause GE deaths and in the US NoV is the second leading cause (8 %) of all-cause GE deaths [51, 78, 79]. In analyses that excluded deaths due to *C. difficile,* NoV contributed 16 % of GE deaths among older adults [51]. These findings are comparable to data from England and Wales that modeled seasonal variations in death according to seasonal variations in laboratory reports (excluding deaths due to *Clostridium difficile)* where NoV accounted for 20 % of older adult deaths due to infectious intestinal disease and 13 % of deaths registered as ‘non-infectious gastrointestinal disease’ [78].

Data from the Netherlands estimated that 14 % of unspecified GE deaths were attributable to NoV among older adults with outbreak-associated increases of approximately 130 % above the mean *monthly* unspecified GE death incidence per 10,000 (>0.06 compared with 0.026) [68]. Based on an attributable proportion of 14 %, this equates to a mean *annual* NoV incidence per 10,000 of 0.04 with increases to >0.10 during outbreaks. While these mean estimates are lower than the estimated rates per 10,000 based on German (0.32) and US (0.20) data, the estimated rates during outbreaks reached a comparable level [51, 63, 68].

One publication extrapolated German surveillance data to the Dutch population to obtain estimated mean age-specific NoV case fatality rate per 1000 (95 % CI) of: 0.09 (0.01–0.27) for those <1 year, 0.00 (0–1.12) for those 1–4 years, 0.00 (0–0.04) for those 5–11 years, 0.09 (0–0.34) for those 12–17 years, 0.03 (0.01–0.06) for those 18–64 years and 0.63 (0.55–0.73) for those ≥65 years [80].

In a Swedish-based study of hospitalized adults with community-onset NoV infection, nearly 8 % of individuals died within 30 days of a NoV-positive sample (e.g., an 8 % 30-day NoV mortality rate) and all deaths occurred among those ≥60 years [55]. Individuals with underlying medical conditions had a higher 30-day mortality rate than those without such conditions (10.5 % compared to 5.3 %). The study authors did not specify the relevant underlying conditions. However, another study by Harris et al., noted that circulatory, respiratory and neoplastic conditions were important contributors to NoV-associated deaths [78].

### Institutional NoV outbreaks

National surveillance data from HI countries have reported that the majority of NoV outbreaks occur in hospitals and LTCFs [31, 32, 35]. NoV GII.4 has been identified in a majority of institutional outbreaks among older adults [32, 35].

#### Nosocomial NoV

NoV outbreaks frequently occur in hospitals and attack rates can be extremely high (up to 60 % of patients and 40 % of staff) [31, 32, 39, 81]. In a study of nearly 40,000 NoV cases captured within the German surveillance system, age ≥70 years was significantly associated with nosocomial (e.g., NoV onset >2 days after hospital admission) NoV infection (risk ratio [RR] = 14.3, 95 % CI 13.5–15.0 for individuals 70–81 years and RR = 13.0, 95 % CI 12.4–13.6 for individuals >81 years compared to those ≤25 years) [82]. The proportion of NoV cases considered to be nosocomial showed a U-shaped curve with the largest proportions in those ≥70 years (65 %) followed by infants <1 year (16 %). Approximately 66 % of all NoV nosocomial infections occurred in elderly adults and the importance of nosocomial infections among this population was more pronounced for NoV than for other routinely surveyed GE pathogens such as rotavirus, *Salmonella* and *Campylobacter*. German surveillance data indicate that 83 % of NoV hospitalized cases among those >74 years originated from institutional settings such as hospitals or LTCFs [32].

#### NoV outbreaks in LTCFs

LTCFs are a common setting for AGE outbreaks (50 % of all AGE outbreaks occurred in LTCFs as reported in a nationwide Australian study) of which 30-80 % were due to NoV, largely driven by NoV GII.4 [15, 31, 35, 38, 83–87]. Within the US and Australia, the annual number of NoV outbreaks was estimated to be 6–8 per 100 LTCFs [38, 83].

Seven studies were identified that pertained exclusively to LTCFs [38, 83, 85–89]. The definition of LTCF, facility characteristics, and resident population differed between studies, however LTCF generally referred to facilities that provided prolonged care for individuals who required daily living and/or nursing care support. None of the 7 identified studies provided the age distribution for the study population and only one [89] provided age-stratified results. NoV outbreaks in LTCF settings were characterized by high levels of person-to-person transmission (in many reports >90 % of such outbreaks were transmitted person-to-person) facilitated through caregiving, close contact between residents and staff, as well as frequent movement of infected individuals within and between facilities [31, 38, 83, 85]. NoV outbreaks in LTCFs were associated with higher attack rates and deaths than other causes of AGE outbreaks [31, 38, 63, 83, 88, 89]. NoV was responsible for 70 % of all viral and bacterial enteric illnesses, 58 % of all hospitalizations, and 27 % of deaths in a review of enteric illness outbreaks in LTCFs [85]. This represents a much higher impact on severe health outcomes among LTCFs compared to other settings (versus 8–17 % of AGE hospitalizations and 8–20 % of GE deaths due to NoV in non-LTCF settings) [45, 51, 68–70, 73, 78].

Table 2 summarizes the attack, case hospitalization and case fatality rates reported for NoV in the identified LTCF studies. Attack rates ranged from 3 to 45 % depending on the study and largely reflected differences in the number reporting/laboratory confirmation and definition of individual outbreaks, facility characteristics, and study population [38, 83, 85–88]. The median attack rate estimates from a global review by Utsumi et al. [86] for residents (45 %) and staff (42 %) included outbreaks with attack rates of 100 % which may overestimate ‘typical’ attack rates. However, these high estimates are similar to GII.4 attack rates among individual wards of nursing homes estimated from the Netherlands [87].

**Table 2:** Attack, case hospitalization and case fatality rates during norovirus -associated outbreaks in long-term care facilities

Author (Ref)	Location	Number of norovirus (NoV) outbreaks	Population included in estimates	NoV-associated outcome^a^		
				Attack rate (%)	Case hospitalization rate (%)	Case fatality rate (%)
Davis [88]	Queensland, Australia	264	Residents and staff	20.3 any NoV-associated illness	1.7	0.5
Kirk [83]	Nationwide, Australia	1136	Residents and staff	--	--	0.3
Rosenthal [38]^b^	Oregon, US	163	Residents	4; All facilities	3.1; All facilities	0.5; All facilities
				6; Nursing facilities	1.8; Nursing facilities	0.5; Nursing facilities
				3; Non-nursing facilities	4.3; Non-nursing facilities	0.4; Non-nursing facilities
Friesma [87]^c^	5–6 local health services, NL	28	Residents	Nursing homes	0.5	1.6
				15.5 GII.4		
				12.8 Non-GII.4		
				Individual wards of nursing homes		
				40.0 GII.4		
				31.1 Non-GII.4		
Greig [85]	Global review	43	Residents	--	4.2	0.7
Utsumi [86]^d^	Global review	23	Residents	45 Residents	6	--
			Staff	42 Staff		

^a^Definition of Nov-associated outcome may have differed across individual studies

^b^Rosenthal et al. [38] defined nursing facility as those institutions providing 24-h nursing care and non-nursing facility otherwise; average attack rates reported

^c^Friesma et al. [87] report NoV outbreaks in both residents and staff but results reported for residents only; median attack rates reported

^d^Utsumi et al. [86] report median attack rates

Case hospitalization rates ranged from 0.5 % [87] to 6 % [86]. The highest median hospitalization rates were reported again in the review by Utsumi et al. which may reflect reporting differences in the primary studies [86]. However, data from a US-based study and a second global review suggest case hospitalization rates range between 2–4 % depending on the sub-population within LTCFs [38, 85]. Considered from a different perspective, of all enteric outbreaks in LTCFs, NoV is responsible for 58 % of hospitalizations [85]. An estimated 34.4 NoV-associated hospitalizations occur annually per 10,000 individuals in LTCFs [88].

Case fatality rates due to NoV in residents of LTCFs ranged from 0.3 % [83] to 1.6 % [87]. The variability in case fatality rate is presumably due to study-specific definitions of NoV-associated deaths, which included broad all-cause mortality during outbreaks in highest estimates obtained in the Netherlands [87].

Trivedi et al. [89] modeled the rates of all-cause hospitalization and mortality during NoV outbreaks compared to non-outbreak periods among LTCF residents (for facilities with ≥1 reported outbreak) within 3 US states. All-cause hospitalization (adjusted RR [aRR] = 1.09; 95 % CI 1.05–1.14) and mortality (aRR = 1.11; 95 % CI 1.05–1.18) rates were significantly increased during NoV outbreak periods. Increased rates were particularly apparent among residents ≥ 90 years (aRR = 1.24; 95 % CI 1.13, 1.37 for all-cause hospitalizations and aRR = 1.28; 95 % CI 1.14, 1.42 for all-cause mortality). The estimated 12 % increase in all-cause hospitalizations and 25 % increase in deaths occurred primarily during the first 2 weeks of outbreaks, with estimates of 1 excess hospitalization for every 4 outbreaks and 1 excess death for every 9 outbreaks.

A few key factors were found to influence NoV risk and/or severity in the LTCF setting which include NoV genotype, age, comorbidity and facility characteristics. NoV GII.4 outbreaks in LTCFs have been associated with more frequent, severe and longer duration of illness compared to non-GII.4 outbreaks [38, 87]. The mean age of NoV cases in LTCFs has been reported to be in the mid-80s, and severe outcomes (including all-cause hospitalizations and mortality) were most frequently reported in the very aged [38, 87, 89]. Facility characteristics, such as level and adequacy ratings of nursing support within LTCFs were associated with the variable risk and severity of NoV outbreaks suggesting that poor underlying health status of residents may influence NoV susceptibility and transmission and inadequate nursing care is an important determinant of NoV-associated mortality [38, 89].

### Estimated number of NoV cases in HI and UMI settings

According to 2013 World Bank estimates, there were approximately 209 million individuals ≥65 years living in HI countries (16 % of total HI population) and 193 million individuals ≥65 years living in UMI countries (8 % of total UMI population).

**Fig. 2:**
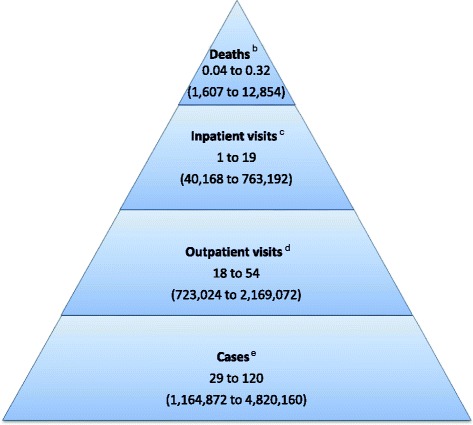
Estimated annual incidence rate per 10,000 (extrapolated number) of norovirus-associated gastroenteritis cases in older adults living in high and upper-middle income countries^a^. ^a^Population based on 2013 World Bank estimate of 401,680,000 individuals aged ≥65 years in high (208,960,000) and upper-middle income (192,720,000) countries. ^b^Annual mortality rate estimates based on following sources: Hall et al. [51], Werber et al. [63], van Asten et al. [68]. ^c^Annual inpatient rate estimates based on following sources: Haustein et al. [69], Lopman et al. [70], van Asten et al. [68]. ^d^Annual outpatient rate estimates based on following sources: Gastanaduy et al. [45], Phillips et al. [42], van Asten et al. [68]. ^e^Annual case rate estimates based on following sources: Werber et al. [63], Bernard et al. [32]; if estimate accounts for incidence data published in de Wit et al. [94] which was published prior to the inclusion dates for this review, the upper incidence rate estimate would be 310 per 10,000 which corresponds to 12,452,080 estimated cases in high and upper middle income countries annually

Figure 2 provides estimated rates of NoV-associated health outcomes among older adults and extrapolated annual cases in HI and UMI countries. The wide range of the estimates reflect the differences in 1) study population; 2) detection and attribution methodology of NoV to GE episodes; and 3) NoV activity level and genotype distribution.

## Discussion

We identified 39 studies from HI and UMI countries that reported information on sporadic and outbreak-associated NoV risk among older adults. Our findings demonstrate a high burden of NoV-associated illness in all ages with the highest burden of severe NoV-associated outcomes occurring among older adults. The incidence of NoV-associated hospitalization in older adults was more frequent, more severe, resulted in longer stays and incurred greater costs than for younger patients [32, 68–70, 72]. Older adults are a key driver of overall NoV-hospitalization rates reported across different settings [8, 33, 82, 90]. Estimated NoV-associated mortality rates were roughly 200 % higher among those ≥65 years compared to <5 year olds. In older adults, NoVs cause approximately 10–20 % of GE hospitalizations, 10–15 % of GE deaths, and ≥0.2 % of all-cause mortality [45, 51, 68, 69, 73, 77–79]. While our review was limited to English-language publications thereby potentially missing relevant manuscripts published in other languages, our review highlights the substantial risk of NoV among institutionalized older adults in HI and UMI countries and supports the adverse impact NoV-associated illness has on healthcare costs and institutional control efforts including a substantial disruption in LTCF staff time and cost (including staff illness) as a result of NoV outbreaks [31, 38, 70, 83–89, 91–93].

We did not identify any community-based incidence data for non-medically attended NoV-associated illness in older adults. Previously published data (2001), however, provide age-specific GE incidence rates (non-pathogen specific) in the general population per 10,000 of: 7400 among those <1 year, 9000 among those 1–4 years, 4810 among those 5–11 years, 1570 among those 12–17 years, 2340 among those 18–64 years and 1940 among ≥65 year olds [94]. The estimated percentage of GE cases positive for Norwalk-like virus or Sapporo-like virus was 16 % (across all ages but within the range of 4–37 % detected in elderly outpatient visits). Thus, it can be roughly estimated that older adults experienced an incidence of 310/10,000 (1940 *0.16) of community-based NoV-associated illnesses representing an approximate percentage increase of 400 to 700 % from the estimated rate of NoV-associated outpatient visits (37–54/10,000) among older adults. While a direct comparison of NoV rates between age groups was not the aim of this review, data suggests that non-medically attended NoV illness and outpatient rates are lower for older adults compared to children <5 years.

We found heightened NoV-associated hospitalizations and deaths among older adults with underlying health conditions which is in agreement with a recent review by Trivedi et al. [95] that identified immunosuppressive conditions to be common among NoV-associated deaths in older adults. The immediate cause of death reported among these individuals included aspiration/pneumonia, sepsis, gastrointestinal and cardiac complications. There is, however, limited reporting of susceptibility to, and clinical course of, NoV among older adults according to underlying health status. Data is also limited on the impact of NoV-associated AGE on daily living, function and quality of life among this population.

The ability to compare or pool study estimates in our review was limited by the variability inherent in the data which reflects different study designs and study populations, inaccuracies in NoV detection/attribution and potentially marked differences in NoV activity over different time periods. Among the publications included in our review, factors of interest were often omitted such as: age distribution, living situation (community or LTCF), health status and/or contributing factors, NoV circulation and genotype, and definition of AGE or GE. Moreover, while much data described AGE outbreaks in healthcare settings, endemic (e.g., cases identified outside of a recognized outbreak setting) rates of NoV illness tended to be less well described. It was difficult to differentiate sporadic (endemic) and outbreak-related NoV risk estimates in studies that reported variable levels of NoV circulation, such as surveillance data or secondary healthcare utilization databases. However it is clear that NoV-associated illnesses and severe outcomes peak during periods of high viral circulation and that NoV outbreaks significantly increase GE and all-cause hospitalizations and mortality among older adults.

One specific source of bias that is well recognized for common non-specific infectious illnesses, is the impact of both under-ascertainment (of illnesses that do not result in a healthcare visit) and under-reporting (correctly diagnosing and reporting NoV cases with non-specific symptoms) [96]. While age-specific (and, ideally, health outcome-specific) NoV multiplication factors are not routinely used, the underestimation of NoV-risk for all ages, including older adults, can be substantial. This is evident by 1) the estimated 400–700 % increase in the NoV incidence rate of community-level illness [94] compared to the estimated rate of NoV-associated outpatient visits among the elderly [42] and, 2) the estimated ≥1000 fold difference in NoV hospitalization rate estimates based on NoV-specific diagnostic codes [71, 72] and model-based indirect estimates that account for undiagnosed NoV cases [69, 70]. Among older adults NoV GII.4 is the most predominant genotype in outbreak, inpatient, LTCFs and community settings, potentially due to a combination of novel GII.4 mutations that emerge (such as the insertion of an amino acid in recent GII.4 strains) and limited immune response among older adults due to immunosenescence [35, 38, 66, 90, 97]. NoV GII.4 has also been identified as the dominant genotype causing illness in children with slightly greater genotype variability (particularly in the GII genogroup) due, perhaps, to less dynamic evolution of non-GII.4 genotypes [8, 11, 90, 97]. Interpretation of NoV risk and genotype distribution across settings is contingent not only upon ascertainment and reporting of individual cases but also upon systematic capture of viral circulation and illness through established surveillance systems. NoV surveillance is largely designed to identify and control outbreaks, and notifiable disease reporting may differentially capture and diagnostically confirm symptomatic infections in closed/semi-closed settings rather than open settings, thereby providing risk estimates and genotype information specific to such settings. NoV-associated illness, hospitalizations and deaths among older adults outside of institutional settings may be missed, resulting in an underestimate of risk and less-than-complete understanding of genotype distribution. Given that older adults seek healthcare for GE less frequently than the very young [98], it may be even more likely that sporadic NoV-associated illness in community-living older adults is underestimated.

Globally, we are experiencing a rapid increase in the relative proportion of older adults due to smaller family size (i.e., fewer children) and longer life expectancy. It is estimated that the proportion of individuals >60 years will double between 2010 and 2050 reaching an estimated 20–30 % in many Organisation for Economic Co-operation and Development (OECD) countries and even 38–40 % in countries such as Japan and Korea [99]. This trend is also projected for the very aged (>80 years) with concomitant increases in the need for long term care (expected to quadruple by 2050 from an estimated 0.2–2 % of total OECD population to 0.8–8 % of total OECD countries population). Preparations are needed for the impact of the ‘age wave’ on the burden of disease caused by common infectious diseases such as NoV [99–101]. Thus, the burden of NoV illness in older adults is likely to increase as the number and proportion of aged, and the very aged (>80 years), increases. While we have provided rough estimates of the absolute number of NoV-associated GE cases in older adults living in HI and UMI countries based on extrapolated annual incidence rates, these figures should be interpreted with caution as the estimates do not account for factors such as country-specific age structure, health status or institutional residence status among older adults.

Several virus-like particles (VLP) NoV vaccines are in development which target key genotypes using a variety of production methods and administration routes [102]. Recent Phase 2 study results of a bivalent (GI and GII.4) VLP NoV vaccine have demonstrated that a candidate vaccine was generally well tolerated with efficacy observed against symptomatic illness among human volunteers vaccinated and subsequently challenged with live NoV [102, 103]. Given the increased risk of NoV-associated hospitalizations and death among older adults, regulatory approval of a safe and effective NoV vaccine which has broad coverage for common and emerging genotypes, that provides durable immunity and is efficacious among older adults, could have a positive impact in this population [104]. NoV vaccination may be a particularly attractive prevention tool for institutionalized older adults, and staff who have contact with them, for whom current control activities have shown limited/variable impact on outbreak duration and attack rates [78, 105]. This has been the case for influenza in which, like NoV, older adults account for approximately 90 % of deaths and 50 % of hospitalizations. Influenza vaccination has resulted in a 77 % reduction in influenza-associated hospitalizations in adults aged ≥50 years [51, 55, 63, 70, 106, 107]. While NoV-associated age-specific mortality and hospitalization rates among the elderly are generally lower than those reported for influenza, NoV estimates can reach a comparable level among the very aged during epidemic NoV seasons (such as 2006/7) [70, 108].

## Conclusion

We described the NoV-associated burden of disease among older adults and highlighted the importance of severe NoV-associated outcomes in this population. We estimated that in HI and UMI countries there are 1.2 to 4.8 million NoV-associated illnesses (accounting for under-ascertainment this estimate could increase to 12.5 million), 723,000 to 2.2 million NoV-associated outpatient visits, 40,000 to 763,000 NoV-associated hospitalizations, and 2000 to 13,000 NoV-associated deaths annually. Older adults living in LTCFs are at increased risk of NoV-associated illness due to frequent outbreaks and severe health outcomes. NoV prevention has the potential to significantly reduce the NoV-associated burden of disease in all ages with perhaps the most impact on severe outcomes in older adults. This potential impact will be heightened in the coming years due to rapid aging of societies and projected increases in aged individuals requiring long term care.

## Abbreviations

AGE: Acute gastroenteritis
aRR: Adjusted risk ratio
ED: Emergency department
GE: Gastroenteritis
HBGAs: Histo-blood group antigens
HI: High income
ICD: International classification of diseases
LTCF: Long-term care facility, LTCFs Long-term care facilities
NoV: Norovirus, NoVs-Noroviruses
OECD: Organisation for Economic Co-operation and Development
RR: Risk ratio
UMI: Upper-middle income
US: United States
